# Effectiveness of COVID-19 booster vaccines against COVID-19-related symptoms, hospitalization and death in England

**DOI:** 10.1038/s41591-022-01699-1

**Published:** 2022-01-14

**Authors:** Nick Andrews, Julia Stowe, Freja Kirsebom, Samuel Toffa, Ruchira Sachdeva, Charlotte Gower, Mary Ramsay, Jamie Lopez Bernal

**Affiliations:** 1 UK Health Security Agency, London, UK; 2 NIHR Health Protection Research Unit in Vaccines and Immunisation, London School of Hygiene and Tropical Medicine, London, UK; 3 NIHR Health Protection Research Unit in Respiratory Infections, Imperial College London, London, UK

**Keywords:** Epidemiology, Infectious diseases

## Abstract

Booster vaccination with messenger RNA (mRNA) vaccines has been offered to adults in England starting on 14 September 2021. We used a test-negative case–control design to estimate the relative effectiveness of a booster dose of BNT162b2 (Pfizer-BioNTech) compared to only a two-dose primary course (at least 175 days after the second dose) or unvaccinated individuals from 13 September 2021 to 5 December 2021, when Delta variant was dominant in circulation. Outcomes were symptomatic coronavirus disease 2019 (COVID-19) and hospitalization. The relative effectiveness against symptomatic disease 14–34 days after a BNT162b2 or mRNA-1273 (Moderna) booster after a ChAdOx1-S (AstraZeneca) and BNT162b2 as a primary course ranged from around 85% to 95%. Absolute vaccine effectiveness ranged from 94% to 97% and was similar in all age groups. Limited waning was seen 10 or more weeks after the booster. Against hospitalization or death, absolute effectiveness of a BNT162b2 booster ranged from around 97% to 99% in all age groups irrespective of the primary course, with no evidence of waning up to 10 weeks. This study provides real-world evidence of substantially increased protection from the booster vaccine dose against mild and severe disease irrespective of the primary course.

## Abstract (web summary)

A test-negative case–control analysis of data from the National Immunisation Management System in England demonstrates significant levels of increased protection against hospitalization or death from COVID with mRNA booster vaccines following a primary two-dose course of either ChAdOx1-S or BNT162b2 vaccines.

## Main

Real-world effectiveness data has demonstrated high levels of short-term protection by COVID-19 vaccines against clinical disease and, more so, against severe outcomes, including hospitalization and death^1–7^. Nevertheless, there is evidence that protection against symptomatic disease wanes over time^8,9^. Booster doses have now been implemented in the United Kingdom and elsewhere in order to combat the rise in COVID-19 cases and the additional threat of the winter 2021 influenza season.

We recently reported that vaccine effectiveness against symptomatic disease peaked in the early weeks after the second dose and then fell to 47.3 (95% confidence interval (CI), 45–49.6) and 69.7 (95% CI, 68.7–70.5) by ≥20 weeks against the Delta variant for ChAdOx1-S (AstraZeneca) and BNT162b (Pfizer-BioNTech)), respectively. Vaccine effectiveness against severe disease outcomes remained high up to 20 weeks after vaccination in most groups; nevertheless, greater waning was seen in older adults and those with underlying medical conditions compared to young, healthy adults^8^.

In the United Kingdom, COVID-19 booster vaccines were introduced on 14 September 2021. Using evidence from the COV-BOOST trial, which demonstrated that the mRNA vaccines provide a strong booster effect with low reactogenicity, regardless of the vaccine given in the primary course, the UK Joint Committee on Vaccination and Immunisation recommended either a BNT162b2 or a half dose (50 µg) of mRNA-1273 (Moderna) vaccine to be given as a booster dose no earlier than 6 months after completion of the primary vaccine course^10,11^. In this initial phase of the UK booster program, the following groups were eligible: all adults >50 years and those 16–49 years with underlying health conditions that put them at higher risk of severe COVID-19, adult carers and adult household contacts (aged ≥16 years) of immunosuppressed individuals and healthcare workers.

In this study, we aimed to estimate the effectiveness of the BNT162b2 and mRNA-1273 booster vaccines against symptomatic disease, hospitalization and death in adults in England. Table 1 outlines the main findings and implications for policy of our study.

**Table 1:** Policy summary

Background	Following evidence of waning protection after a primary course of COVID-19 vaccines, booster doses are now being offered in the United Kingdom and elsewhere. There is limited evidence of the effectiveness of booster doses.
Main findings and limitations	We observed a substantial increase in protection against symptomatic COVID-19 disease with the Delta variant after a booster dose of an mRNA vaccine irrespective following a primary course of two doses of either BNT162b2 or ChAdOx1-S. There was limited waning by 10 or more weeks after vaccination. A longer interval between primary course and booster vaccination was associated with small improvements in vaccine effectiveness.
	Very high levels of protection (97-99%) were seen against hospitalization or death with a BNT162b2 booster, with no evidence of waning up to 9 weeks after the booster.
	This is an observational study, and there may be residual confounding that could not be accounted for. There may also be misclassification due to imperfect sensitivity of PCR testing.
Policy implications	COVID-19 booster vaccination programs are likely to result in substantial reductions in cases, hospitalizations and deaths with COVID-19. There is some benefit of a longer interval between primary course and booster vaccination, but this needs to be balanced with reduced protection among those who have only received two doses.

## Results

### Descriptive statistics and characteristics

From 13 September 2021 to 5 December 2021, there were a total of 893,845 eligible test results for individuals aged ≥18 years with a test date within 10 days of their symptom onset date and a link to the National Immunisation Management System (NIMS), with a 91.04% match rate. Of these eligible participants, 278,096 (31.1%) were unvaccinated, 223,198 received ChAdOx1-S 175 days after a second dose and 171,079 received BNT162b2 175 days after a second dose. Of those who had received a booster dose, 89,019 received a ChAdOx1-S primary course and 132,453 received a BNT162b2 primary course. Of the 343,955 positive cases included in the analysis, 4,377 (1.27%) were hospitalized for any reason (excluding injuries) within 14 days of the test. A description of the eligible tests is given in Supplementary Table 1.

### Vaccine effectiveness for symptomatic disease

**Fig. 1:**
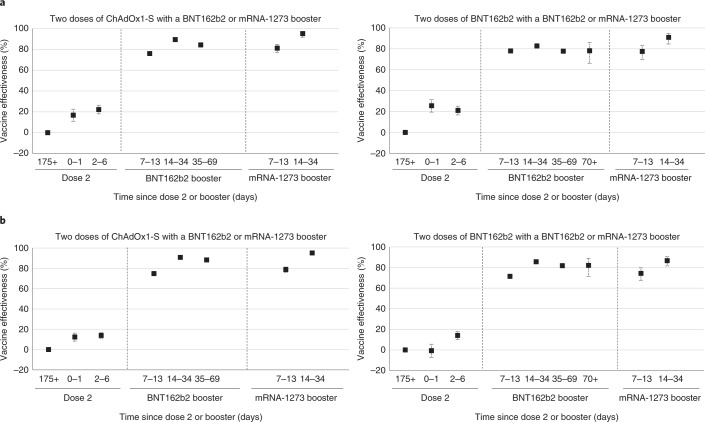
Estimates of vaccine effectiveness against symptomatic disease after booster according to primary course. **a**,**b**, Vaccine effectiveness estimates (95% CI) against symptomatic disease in time intervals after booster according to primary course in individuals aged 18–49 years (**a**) 50 years and older (**b**). Dose 2 was received at 175 days as the baseline.

**Extended Data Fig. 1:**
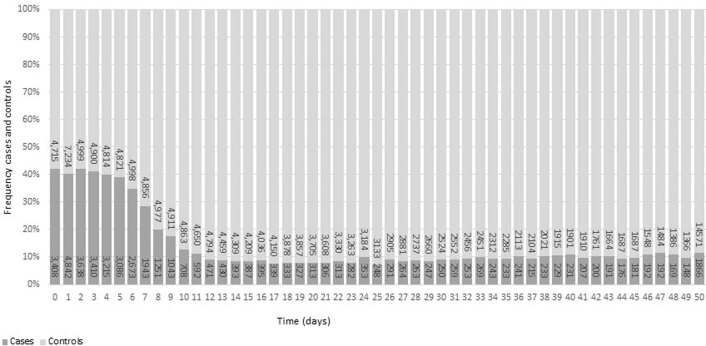
Distribution (%) and counts of cases and controls by interval from booster to onset.

An overall effect on the proportion of cases and controls was seen from around day 7 after the booster dose and stabilized at day 11 (Extended Data Fig. 1). In individuals aged 18 to 49 years where the primary course was ChAdOx1-S vaccine, relative to those who had received only two doses, effectiveness against symptomatic disease peaked at 14–35 days after the BNT162b2 booster at 89.6% (95% CI, 88.6–90.4) and 95.3% (95% CI, 91.8–97.3) after the mRNA-1273 booster (Table 2 and Fig. 1). In individuals where BNT162b2 was the primary course, relative vaccine effectiveness 14-34 days a BNT162b2 booster was 82.8% (81.8-83.7) and after a mRNA-1273 booster 90.9% (84.5–94.7). Relative vaccine effectiveness with the BNT162b2 booster decreased slightly in the 35- to 69-day and ≥70-day periods (later follow-up was not available for mRNA-1273). The same analysis in individuals aged 50 years and older gave similar results (Table 2 and Fig. 1).

**Table 2:** Vaccine effectiveness against symptomatic disease for the BNT162b2 (Pfizer-BioNTech) and mRNA-1273 (Moderna) booster vaccines in England by age group

Age group (years)	Primary course (≥175 days after dose 2)	Booster	Interval since booster (days)	Controls	Cases	rVE (≥175 days after dose 2 baseline) (95% CI)	rVE (dose 3: 2–6 days after booster baseline) (95% CI)	VE (unvaccinated base) (95% CI)
18-49	Unvaccinated			125,353	126,940			
	ChAdOx1-S	None		61,022	45,988	Baseline		44.7 (43.7–45.6)
	ChAdOx1-S	Any	0–1	2,111	1,407	16.8 (10.8–22.4)		54.5 (51.2–57.6)
	ChAdOx1-S	Any	2–6	3,947	2,467	22.3 (18.0–26.3)	Baseline	57.2 (54.8–59.4)
	ChAdOx1-S	BNT162b2	7–13	3,984	736	76.1 (74.1–78)	69.3 (66.2–72.0)	86.8 (85.7–87.9)
	ChAdOx1-S	BNT162b2	14–34	7,174	561	89.6 (88.6–90.4)	86.6 (85.2–87.9)	94.3 (93.8–94.8)
	ChAdOx1-S	BNT162b2	35–69	2,927	319	84.4 (82.4–86.1)	79.9 (77.2–82.3)	91.6 (90.5–92.5)
	ChAdOx1-S	mRNA-1273	7–13	635	98	81.3 (76.8–84.9)	75.9 (70.0–80.6)	89.7 (87.2–91.7)
	ChAdOx1-S	mRNA-1273	14–34	342	13	95.3 (91.8–97.3)	93.9 (89.4–96.5)	97.4 (95.5–98.5)
	BNT162b2	None		79,181	29,489	Baseline		65.3 (64.7–65.9)
	BNT162b2	Any	0–1	2,800	839	25.6 (19.4–31.3)		73.7 (71.5–75.7)
	BNT162b2	Any	2–6	6,186	2,046	21.0 (16.7–25.1)	Baseline	71.8 (70.3–73.3)
	BNT162b2	BNT162b2	7–13	8,797	825	77.9 (76.2–79.5)	72 (69.5–74.4)	92.1 (91.5–92.7)
	BNT162b2	BNT162b2	14–34	20,595	1,614	82.8 (81.8–83.7)	78.2 (76.5–79.7)	93.9 (93.6–94.2)
	BNT162b2	BNT162b2	35–69	16,703	1,707	77.7 (76.4–78.9)	71.7 (69.6–73.7)	92.1 (91.6–92.5)
	BNT162b2	BNT162b2	≥70	194	22	78.1 (65.8–86)	72.3 (56.6–82.3)	92.0 (87.5–94.8)
	BNT162b2	mRNA-1273	7–13	397	49	77.4 (69.6–83.3)	71.4 (61.3–78.9)	91.9 (89.0–94.0)
	BNT162b2	mRNA-1273	14–34	290	14	90.9 (84.5–94.7)	88.5 (80.3–93.3)	96.7 (94.4–98.1)
≥50	Unvaccinated			10,322	15,481			
	ChAdOx1-S	None		55,808	60,380	Baseline		39.4 (37.4–41.3)
	ChAdOx1-S	Any	0–1	4,284	4,212	12.3 (8.3–16.2)		46.9 (44.0–49.6)
	ChAdOx1-S	Any	2–6	7,924	7,762	13.9 (10.9–16.8)	Baseline	47.7 (45.3–50.0)
	ChAdOx1-S	BNT162b2	7–13	8,887	2,514	74.8 (73.6–75.9)	70.7 (69.1–72.3)	84.7 (83.8–85.5)
	ChAdOx1-S	BNT162b2	14–34	16,437	1,691	90.8 (90.3–91.3)	89.4 (88.7–90.0)	94.4 (94.1–94.7)
	ChAdOx1-S	BNT162b2	35–69	5,432	703	88.3 (87.3–89.2)	86.4 (85.2–87.5)	92.8 (92.2–93.4)
	ChAdOx1-S	mRNA-1273	7–13	1,275	317	78.9 (76.1–81.4)	75.5 (72.2–78.5)	87.2 (85.4–88.7)
	ChAdOx1-S	mRNA-1273	14–34	770	44	95.2 (93.4–96.4)	94.4 (92.4–95.9)	97 (96.0–97.8)
	BNT162b2	None		38,673	23,736	Baseline		61.2 (59.8–62.5)
	BNT162b2	Any	0–1	2,753	1,792	−0.7 (−7.3 to 5.5)		61 (58.2–63.5)
	BNT162b2	Any	2–6	6,474	3,747	14 (10.1–17.8)	Baseline	66.6 (64.8–68.2)
	BNT162b2	BNT162b2	7–13	9,094	1,812	71.4 (69.8–72.9)	66.7 (64.5–68.8)	88.9 (88.2–89.5)
	BNT162b2	BNT162b2	14–34	22,158	2,352	85.6 (84.9–86.3)	83.3 (82.2–84.2)	94.4 (94.1–94.7)
	BNT162b2	BNT162b2	35–69	15,931	2,119	81.9 (80.8–82.8)	78.9 (77.5–80.2)	92.9 (92.5–93.3)
	BNT162b2	BNT162b2	≥70	165	20	82.1 (71.3–88.8)	79.2 (66.6–87.0)	93 (88.8–95.6)
	BNT162b2	mRNA-1273	7–13	440	86	74.4 (67.6–79.7)	70.2 (62.2–76.5)	89.9 (87.3–92)
	BNT162b2	mRNA-1273	14–34	374	39	86.8 (81.5–90.5)	84.6 (78.5–89.0)	94.8 (92.7–96.3)

rVE, relative vaccine effectiveness compared to dose 2 (either ≥175 days after dose 2 with no booster or ≥175 days after dose 2 and 2–6 days after booster); VE, vaccine effectiveness compared to zero doses.

**Extended Data Fig. 2:**
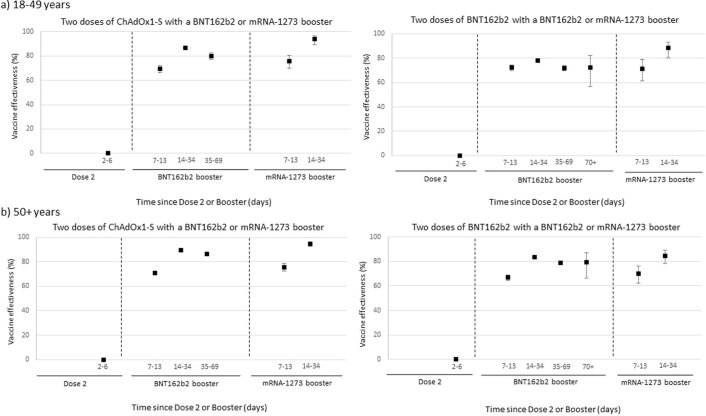
Vaccine Effectiveness estimates (95% CI) against symptomatic disease in time intervals post booster according to primary course in individuals aged a) 18 to 49 years b) 50 years and over: Dose 3 at 2-6 days as baseline.

**Extended Data Fig. 3:**
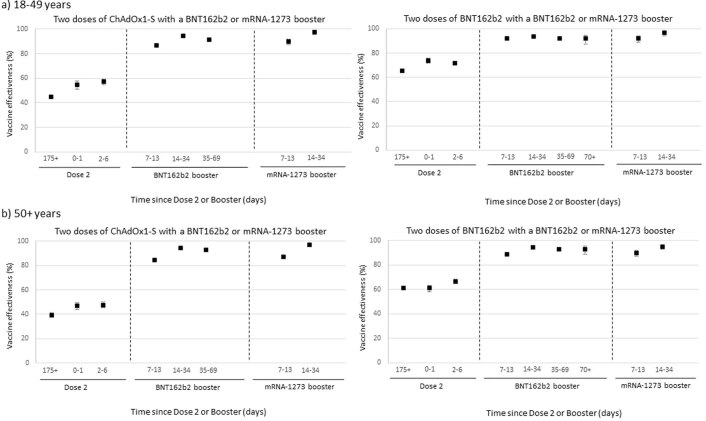
Vaccine Effectiveness estimates (95% CI) against symptomatic disease in time intervals post booster according to primary course in individuals aged a) 18 to 49 years b) 50 years and over: Unvaccinated baseline.

In the secondary analysis, which used the 2- to 6-day period after the booster dose as the baseline, results were similar to the primary analysis (Table 2 and Extended Data Fig. 2). In the analysis using the unvaccinated individuals as the baseline, the booster dose was associated with an absolute vaccine effectiveness from 14 to 34 days after a BNT162b2 booster of 94.4% (95% CI, 94.1–94.7) following either a ChAdOx1-S or BNT162b2 primary course in individuals 50 years and older. With an mRNA-1273 booster, absolute vaccine effectiveness was 97.0 (95% CI, 96.0–97.8) after a ChAdOx1-S primary course and 94.8% (95% CI, 92.7–96.3) after a BNT162b2 primary course (Table 3 and Extended Data Fig. 3).

**Table 3:** Vaccine effectiveness against hospitalization for the BNT162b2 (Pfizer-BioNTech) booster vaccines in England by age group

Age group (years)	Primary course (≥175 days after dose 2)	Booster	Interval since booster (days)	Controls	Cases	VE (unvaccinated base) (95% CI)
18-49	Unvaccinated			111,292	1,366	Baseline
	ChAdOx1-S	None		42,032	171	85.7 (82.9–88.1)
	ChAdOx1-S	Any	0–1	1,244	5	89.2 (73.7–95.5)
	ChAdOx1-S	Any	2–6	2,181	6	93.0 (84.2–96.9)
	ChAdOx1-S	BNT162b2	7–13	2,498	6	93.8 (86.1–97.3)
	ChAdOx1-S	BNT162b2	14–34	4,284	4	97.5 (93.3–99.1)
	ChAdOx1-S	BNT162b2	35–69	1,279	2	94.7 (78.7–98.7)
	BNT162b2	None		70,347	72	94.8 (93.3–96.0)
	BNT162b2	Any	0–1	2,398	6	89.9 (77.3–95.5)
	BNT162b2	Any	2–6	5,275	3	97.8 (93.1–99.3)
	BNT162b2	BNT162b2	7–13	7,552	2	98.9 (95.8–99.7)
	BNT162b2	BNT162b2	14–34	16,531	5	98.8 (97.2–99.5)
	BNT162b2	BNT162b2	35–69	8,697	2	99.1 (96.4–99.8)
≥50	Unvaccinated			9,093	719	Baseline
	ChAdOx1-S	None		41,992	807	85.6 (83.7–87.3)
	ChAdOx1-S	Any	0–1	2,877	47	87.3 (82.7–90.7)
	ChAdOx1-S	Any	2–6	5,151	47	93.9 (91.6–95.5)
	ChAdOx1-S	BNT162b2	7–13	6,029	24	97.6 (96.3–98.4)
	ChAdOx1-S	BNT162b2	14-34	9,664	14	99.2 (98.6–99.5)
	ChAdOx1-S	BNT162b2	35–69	2,130	1	99.7 (98.1–100.0)
	BNT162b2	None		36,093	424	92.1 (90.8–93.2)
	BNT162b2	Any	0–1	2,469	27	93.0 (89.4–95.3)
	BNT162b2	Any	2–6	5,743	43	95.5 (93.7–96.7)
	BNT162b2	BNT162b2	7–13	7,843	21	98.5 (97.6–99.0)
	BNT162b2	BNT162b2	14–34	17,393	45	98.6 (98.0–99.0)
	BNT162b2	BNT162b2	35–69	8,424	19	98.7 (97.8–99.2)

### Vaccine effectiveness for hospitalization and death

High levels of protection were also seen against hospitalization in both age groups. In individuals aged 50 years and older, the vaccine effectiveness 14-34 days after a BNT162b2 booster dose, relative to unvaccinated individuals, was 99.2% (95% CI, 98.6–99.5) when the primary course was ChAdOx1-S and 98.6% (95% CI, 98.0–99.0) when BNT162b2 was used as the primary course.

**Fig. 2:**
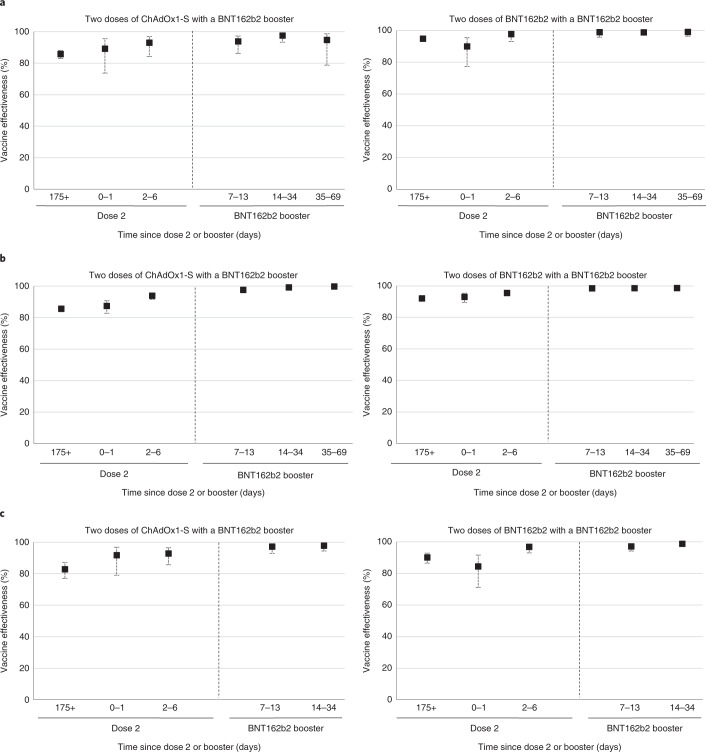
Vaccine effectiveness estimates in time intervals after booster according to primary course against hospitalization or death. **a**–**c**, Vaccine effectiveness estimates (95% CI) in time intervals after booster according to primary course against hospitalization in individuals aged 18–49 years (**a**) and 50 years and older (**b**) and against death in individuals aged 50 years and older (**c**). Unvaccinated individuals served as the baseline.

A similarly high level of protection was seen in the younger age group, with a vaccine effectiveness estimate of 97.5% (95% CI, 93.3–99.1) when the primary course was ChAdOx1-S and 98.8% (95% CI, 97.2–99.5) when BNT162b2 was used as the primary course (Table 3 and Fig. 2). There was little evidence of any waning in vaccine effectiveness against hospitalization up to 69 days after the booster.

Vaccine effectiveness against death in individuals 50 years and older 14–34 days after a BNT162b2 booster dose relative to the unvaccinated was 97.8 (95% CI, 94.4–99.1) after a ChAdOx1-S primary course and 98.7% (95% CI, 97.4–99.4) when the primary course was BNT162b2 (Table 4 and Fig. 2)

**Table 4:** Vaccine effectiveness against death for the BNT162b2 (Pfizer-BioNTech) booster vaccine in England in individuals aged 50 years and older

Primary course (≥175 days after dose 2)	Booster	Interval since booster (days)	Controls	Cases	VE (unvaccinated base) (95% CI)
Unvaccinated			7,470	107	Baseline
ChAdOx1-S	None		25,641	191	82.8 (76.9–87.2)
ChAdOx1-S	Any	0–1	1,476	5	91.7 (79.0–96.7)
ChAdOx1-S	Any	2–6	2,610	10	92.8 (85.7–96.4)
ChAdOx1-S	BNT162b2	7–13	2,956	5	97.2 (92.9–98.9)
ChAdOx1-S	BNT162b2	14–34	3,716	5	97.8 (94.4–99.1)
ChAdOx1-S	BNT162b2	35–69	302	0	
BNT162b2	None		30,263	127	90.2 (86.5–92.8)
BNT162b2	Any	0–1	1,888	13	84.4 (71.1–91.6)
BNT162b2	Any	2–6	4,298	7	96.9 (93.0–98.6)
BNT162b2	BNT162b2	7–13	5,775	10	97.1 (94.1–98.5)
BNT162b2	BNT162b2	14–34	11,286	9	98.7 (97.4–99.4)
BNT162b2	BNT162b2	35–69	2,063	1	99.2 (94.2–99.9)

### Interval between dose 2 and the booster dose

**Extended Data Fig. 4:**
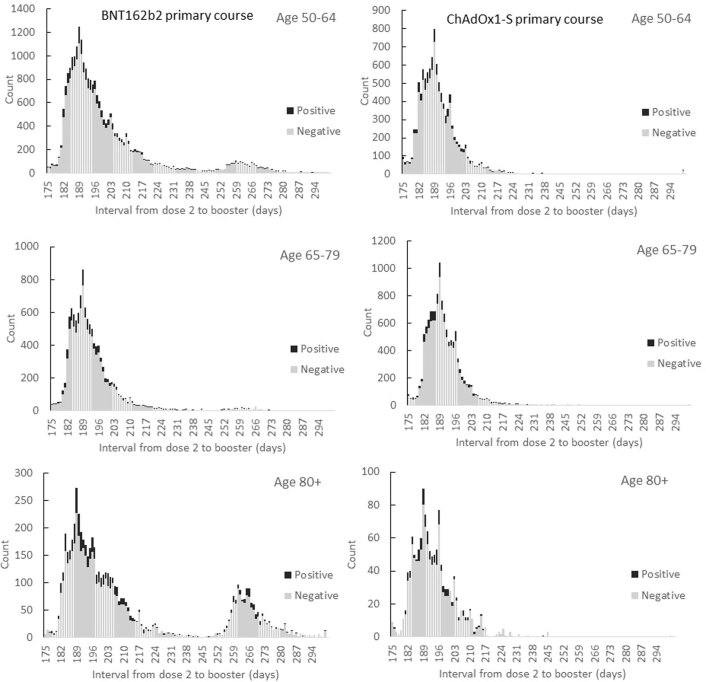
The distribution of intervals between dose 2 and the booster dose for cases and controls by age group and manufacturer.

After assessing the distribution of intervals between dose 2 and the booster dose for cases and controls by age group and manufacturer, the interval between dose 2 and the booster was split into three periods: 25–29, 30–34 and 35 or more weeks (Extended Data Fig. 4). Due to the roll out of the vaccine program, there were more individuals who had received a second dose of BNT162b2 at an earlier time point; therefore, the majority of the individuals who had the longest interval between dose 2 and the booster had a BNT162b2 primary course. Analyses by interval between dose 2 and dose 3 were thus restricted to those who received BNT162b2 as the primary course.

A shorter interval between dose 2 and the booster of 25–29 weeks compared to the baseline interval of 35 weeks or more was associated with an increased adjusted odds ratio of 1.54 (95% CI, 1.35–1.76) for becoming a symptomatic case. This was also seen in the 30- to 34-week interval, with an adjusted odds ratio of 1.32 (1.12–1.56). Although remaining high, the adjusted vaccine effectiveness estimates decreased from 95.6% (95% CI, 94.9–96.1) in the 35 weeks or more interval to 93.2% (95% CI, 92.8–93.6) in the shortest interval between dose 2 and the booster (Supplementary Table 2). A test for the interaction effect of age was not significant (*P* = 0.15).

## Discussion

This study provides evidence of a substantial increase in protection against symptomatic COVID-19 disease after a booster dose of BNT162b2 or mRNA-1273 vaccine during the period when the Delta variant was the dominant strain in the United Kingdom. Very high levels of protection were seen against hospitalization or death with a BNT162b2 booster. Vaccine effectiveness of a booster dose was very similar irrespective of the vaccine used in the primary course. A longer interval between dose 2 and the booster doses was associated with small improvements in vaccine effectiveness^12^.

These findings suggest that the booster offers very high levels of protection against mild and severe disease. Although a small amount of waning in protection against symptomatic disease is seen from 10 weeks after the booster, there is no clear evidence of waning against severe disease up to 10 weeks after the booster. Given the recent deployment of the booster program in the United Kingdom, further follow-up is needed to understand how protection changes longer term against both mild and severe disease. The slightly lower relative VE estimates of the booster in individuals with BNT162b2 as a primary course compared to the ChAdOx1-S in the primary analysis is due to the different baseline with higher VE after two doses of BNT162b2 as compared to ChAdOx1-S (ref. ^8^). When using unvaccinated controls, there was little difference in observed vaccine effectiveness of the booster dose with either primary course. We also observed a peak in testing at day 1 after the booster dose, which is likely to be reactogenicity effects so shortly after the vaccine, as has been reported previously^13^. The improved vaccine effectiveness with a longer interval between dose 2 and the booster suggests that there will be some benefit in delaying booster doses. Nevertheless, this improvement was only small and has to be balanced with the reduced protection among those who have received just two doses (where protection may have waned), compared to protection from the booster even with a relatively short interval. This finding was also similar to the reduced effectiveness among those who had a shorter interval between doses 1 and 2 (refs. ^8,14^). Furthermore, similar findings are also seen with history of prior infection, whereby a longer interval between infection and vaccination was associated with increased protection^15^.

In Israel, a booster program began in July 2021. Bar-On et al. reported an adjusted rate ratio of 11.3 (10.4–12.3) against confirmed infection in booster dose recipients compared to those who received only two doses (equivalent to relative vaccine effectiveness of 91.2%)^16^. This is slightly higher than the relative vaccine effectiveness that we report, which could reflect lower two-dose vaccine effectiveness in the comparison group in Israel, where a greater degree of waning has previously been reported^9,17,18^. Even greater protection has been reported in Israel against severe disease^16,19^. We were unable to find other studies reporting vaccine effectiveness of a third dose when ChAOx1-S was used as the primary course or when mRNA-1273 was used as the booster.

This is an observational study with a number of possible biases and should be interpreted with caution. The imperfect sensitivity of PCR testing could cause misclassification of both cases and controls in a test-negative case–control analysis, which could attenuate vaccine effectiveness estimates. Many individuals will also have been previously infected, so the vaccine effectiveness measured is in the context of a population in which many might have already had natural exposure. We adjust for measured confounders, but there may be residual confounding that we could not account for. Nevertheless, the similarity of the vaccine effectiveness estimates using those with two doses and no booster as the baseline and using the 2- to 6-day period after booster as the baseline suggests that residual confounding is small. Use of individuals who are unvaccinated as a comparator to obtain absolute effectiveness is most susceptible to residual confounding, as the unvaccinated population may differ in many ways from those who have had vaccine doses, which could lead to underestimation of vaccine effectiveness^8^. Despite this potential underestimation, using the unvaccinated comparator, the absolute vaccine effectiveness estimates were over 93%. Due to small numbers, we were only able to report the early effects of the booster program, and it is not yet clear how long protection against COVID-19 following booster vaccination will last.

For the analysis by interval between dose 2 and dose 3, it should be noted that those who had a longer interval between dose 2 and dose 3 are likely to have had a shorter interval between dose 1 and dose 2. As these variables are colinear, it is not possible to adjust for interval between dose 1 and dose 2. In these analyses, we were unable to report on the half dose (50 µg) of mRNA-1273 vaccine due to low numbers, as the majority of booster doses given in this period were BNT162b2. We were unable to assess the vaccine effectiveness in all those targeted for a booster dose, such as individuals with underlying health conditions, adult carers and adult household contacts of immunosuppressed individuals, due to small numbers and difficultly identifying these individuals with the dataset.

Our study provides real-world evidence of substantially increased protection from the booster dose against symptomatic disease and hospitalization in those aged ≥50 years irrespective of which primary course was received. This finding indicates that a high level of protection was achieved among older adults, who are more vulnerable to severe COVID-19. This protection will be important in the 2021–2022 winter period, when COVID-19 is likely to co-circulate alongside other respiratory viruses, including seasonal influenza virus.

## Methods

### Study design

We used a test-negative case–control design to estimate vaccine effectiveness of a booster dose of BNT162b2 vaccine against PCR-confirmed symptomatic disease and hospitalization. We compared vaccination status in symptomatic adults ≥18 years with PCR-confirmed severe acute respiratory syndrome coronavirus 2 (SARS-CoV-2) infection with the vaccination status in individuals who reported symptoms but had a negative SARS-CoV-2 PCR test result. Because an mRNA-1273 vaccine, as a primary course, was not made available until later in the vaccine program, insufficient time had elapsed for a booster dose to be indicated in this group. The study protocol is available in the Supplementary Appendix.

### Ethical approval

Surveillance of COVID-19 testing and vaccination was undertaken under Regulation 3 of The Health Service (Control of Patient Information) Regulations 2002 to collect confidential patient information (www.legislation.gov.uk/uksi/2002/1438/regulation/3/ made) under Sections 3(i) (a) to (c), 3(i)(d) (i) and (ii) and 3(3). The study protocol was subject to an internal review by the Public Health England Research Ethics and Governance Group and was found to be fully compliant with all regulatory requirements. As no regulatory issues were identified and ethical review is not a requirement for this type of work, it was decided that a full ethical review would not be necessary.

### Data sources

#### Vaccination data

NIMS contains demographic information on the entire population of England for individuals who are registered with a general practitioner in England, and this information is used to record all COVID-19 vaccinations. These data were accessed on 14 December 2021. The information used from NIMS included all dates of COVID-19 vaccination and vaccine manufacturer for each dose. Demographic data such as sex, date of birth, ethnicity and residential address were extracted. Addresses were used to determine index of multiple deprivation quintile and were also linked to Care Quality Commission-registered care homes using the unique property reference number. NIMS also contained data on geography (National Health Service (NHS) region), risk groups status, clinically extremely vulnerable and health/social care worker.

Booster doses were identified as being a third dose 175 days or more after a second dose and given after 13 September 2021. Individuals with four or more doses of vaccine, a mix of vaccines in their primary schedule or less than 19 days between their first and second dose were excluded.

#### COVID-19 testing data

SARS-CoV-2 PCR testing in the United Kingdom is undertaken by hospital and public health laboratories, as well as by community testing with the use of drive through or at-home testing, which is available to anyone with symptoms consistent with COVID-19, contacts of a confirmed case, care home staff and residents or anyone who has self-tested positive using a lateral flow device. Initially, data on all positive and negative test results for the period 8 December 2020 to 5 December 2021 were extracted for individuals aged ≥18 years (as of 31 August 2021). Any negative results of tests taken within 7 days of a previous negative test result, or where symptoms were recorded, with symptoms within 10 days of symptoms for a previous negative test result were dropped, as these results likely represent the same episode. Negative test results taken within 21 days before a positive test result were also excluded, as these results are likely to be false negative. Positive and negative test results within 90 days of a previous positive test result were also excluded. Participants contributed a maximum of one randomly chosen negative test result in the follow-up period. Data were restricted to persons who had reported symptoms and gave an onset date. Only persons who had undergone testing within 10 days after symptom onset were included in order to account for reduced sensitivity of PCR testing beyond this period. A small number of positive samples for which sequencing was done and they were found not to be the Delta variant were excluded. Finally, only samples taken from 13 September 2021 (week 37, 2021) were retained for analysis.

Testing data were linked to NIMS on 14 December 2021 using a combination of NHS number (a unique identifier for each person receiving medical care in the United Kingdom), date of birth, surname, first name and postcode using deterministic linkage with >95.5% uniqueness. The NIMS denominator file included information on potential confounding variables related to targeted populations.

#### Hospitalizations

Testing data were linked to the Emergency Care Dataset to assess vaccine effectiveness against hospitalization using data extracted on 15 December 2021. We included emergency care attendances among symptomatic cases within 14 days of the positive test result, which were not injury related and resulted in an inpatient admission. The Emergency Care Dataset includes hospital admissions through NHS emergency departments in England, but not elective admissions. Only first attendances in the 14-day period were selected if a person had more than one admission for emergency care. Data were extracted on 15 December 2021, with cases included if tested by 26 November 2021 to allow for lags in hospitalization.

Data management and linkage were carried out using Microsoft SQL Server Management Studio 18.

### Statistical analysis

Analysis was by logistic regression with the PCR test result as the dependent variable, where those testing positive were cases and those testing negative were controls. Vaccination status was included as an independent variable and effectiveness defined as 1 odds of vaccination in cases/odds of vaccination in controls.

Vaccine effectiveness was adjusted in logistic regression models for age (5-year bands), sex, index of multiple deprivation (quintile), ethnic group, care-home residence status, geographic region (NHS region), period (calendar week of onset), health and social care worker status, clinical risk group status, clinically extremely vulnerable, severely immunosuppressed and previously testing positive. These factors were all considered potential confounders and thus included in all models. To assess the importance of previously testing positive, a sensitivity analysis was done excluding those previously testing positive for the comparison of the booster vaccine to unvaccinated.

Analyses were stratified by which primary doses had been received (ChAdOx1-S or BNT162b2), and any mixed primary courses were excluded.

Vaccine effectiveness against symptomatic disease was assessed for each primary course of vaccine in intervals in days after the booster dose. The BNT162b2 and mRNA-1273 booster doses were combined in the 0- to 1-day and 2- to 6-day postbooster periods. Subsequent periods (7–13, 14–34, 35–69 and ≥70 days) were analyzed separately. There were insufficient data in the last two periods for the mRNA-1273 booster.

Vaccine effectiveness against hospitalization and death was assessed in the combined 0–1 and 2–6 days after the booster and in the 7–13, 14–34 and 35–69 days after the BNT162b2 booster vaccine. All analysis were stratified by 18–49 years and ≥50 years, apart from the death analysis, which was only reported for individuals 50 years and older due to small numbers in <50 year olds.

In the primary analysis, those who had received the booster were compared to individuals who had received two primary doses with at least 175 days before the onset but with no booster dose recorded. In secondary analyses, we also compared to individuals who were completely unvaccinated and the 2- to 6-day period after the booster was received. The 2- to 6-day period was selected after plotting the data on case and control numbers after the booster dose and to avoid days 0 and 1 after the booster, when vaccine reactogenicity may affect the case–control ratio (Fig. 1). The analyses comparing to two doses or the 2- to 6-day postbooster period measured relative effectiveness to two doses, whereas the comparison to individuals who were unvaccinated measured absolute effectiveness of two doses and a booster. In the analysis comparing to individuals who were unvaccinated, we also assessed the remaining effectiveness of two doses at least 175 days (25 weeks) after the second dose.

Among individuals who received BNT162b2 as their primary course, an additional analysis was undertaken estimating the odds of testing positive in shorter intervals between dose 2 and the booster (25–29 and 30–34 weeks) relative to the longest interval (35 or more weeks). A test for the interaction effect of age was also performed. Vaccine effectiveness compared to individuals who were unvaccinated was also stratified by the interval between dose 2 and the booster.

### Reporting Summary

Further information on research design is available in the Nature Research Reporting Summary linked to this article.

## Online content

Any methods, additional references, Nature Research reporting summaries, source data, extended data, supplementary information, acknowledgements, peer review information; details of author contributions and competing interests; and statements of data and code availability are available at 10.1038/s41591-022-01699-1.

## Supplementary information

Supplementary Information Supplementary Tables 1 and 2 and Appendix.

Reporting Summary

Supplementary Table Study protocol.

## Data Availability

No additional data available. Data cannot be made publicly available for ethical and legal reasons, that is public availability would compromise patient confidentiality as data tables list single counts of individuals rather than aggregated data. Databases used in this study include NIMS, Unified Sample Database and the Emergency Care Dataset.

## Code availability

Model fitting code is available on our GitHub site (https://github.com/UKHSAGithub/StataCode.git).
