# Supplementary material for: Effectiveness of COVID-19 booster vaccines against COVID-19-related symptoms, hospitalization and death in England
Source: Nat Med. 2022 Jan 14;28(4):831–7. doi: 10.1038/s41591-022-01699-1 (PMC9018410; doi:10.1038/s41591-022-01699-1)
Supplement: Supplementary file 1 — Supplementary Tables 1 and 2 and Appendix. [file 41591_2022_1699_MOESM1_ESM.pdf]

nature portfolio

https://doi.org/10.1038/s41591-022-01699-1

Supplementary information

Effectiveness of COVID-19 booster vaccines against COVID-19-related symptoms, hospitalization and death in England

In the format provided by the authors and unedited

Supplementary table 1: Descriptive characteristics of positive and negative test results in individuals tested for SARS-CoV-2 in England for the study population. *

				Overall		Positive		Negative	
				n	%	n	%	n	%
	**Test Result**			**893,845**	**100.0%**	**343,955**	**38.5%**	**549,890**	**61.5%**
**Vaccination Status and intervals after vaccine****	**Primary vaccine**	**Booster**	**Days post vaccine**						
	Unvaccinated			278,096	**31.1%**	142,421	**41.4%**	135,675	**24.7%**
	ChAdOx1-S	None	175+	223,198	**25.0%**	106,368	**30.9%**	116,830	**21.2%**
	BNT162b2	None	175+	171,079	**19.1%**	53,225	**15.5%**	117,854	**21.4%**
	ChAdOx1-S	Any	0-1	12,014	**1.3%**	5619	**1.6%**	6,395	**1.2%**
	ChAdOx1-S	Any	2-6	22,100	**2.5%**	10,229	**3.0%**	11,871	**2.2%**
	ChAdOx1-S	BNT162b2	7-13	16,121	**1.8%**	3,250	**0.9%**	12,871	**2.3%**
	ChAdOx1-S	BNT162b2	14-34	25,863	**2.9%**	2,252	**0.7%**	23,611	**4.3%**
	ChAdOx1-S	BNT162b2	35-69	9,381	**1.0%**	1,022	**0.3%**	8,359	**1.5%**
	ChAdOx1-S	BNT162b2	70+	27	**0.0%**	7	**0.0%**	20	**0.0%**
	ChAdOx1-S	mRNA-1273	7-13	2,325	**0.3%**	415	**0.1%**	1,910	**0.3%**
	ChAdOx1-S	mRNA-1273	14-34	1,169	**0.1%**	57	**0.0%**	1,112	**0.2%**
	ChAdOx1-S	mRNA-1273	35-69	19	**0.0%**	4	**0.0%**	15	**0.0%**
	BNT162b2	Any	0-1	8,184	**0.9%**	2,631	**0.8%**	5,553	**1.0%**
	BNT162b2	Any	2-6	18,453	**2.1%**	5,793	**1.7%**	12,660	**2.3%**
	BNT162b2	BNT162b2	7-13	20528	**2.3%**	2637	**0.8%**	17,891	**3.3%**
	BNT162b2	BNT162b2	14-34	46719	**5.2%**	3966	**1.2%**	42,753	**7.8%**
	BNT162b2	BNT162b2	35-69	36460	**4.1%**	3826	**1.1%**	32,634	**5.9%**
	BNT162b2	BNT162b2	70+	401	**0.0%**	42	**0.0%**	359	**0.1%**
	BNT162b2	mRNA-1273	7-13	972	**0.1%**	135	**0.0%**	837	**0.2%**
	BNT162b2	mRNA-1273	14-34	717	**0.1%**	53	**0.0%**	664	**0.1%**
	BNT162b2	mRNA-1273	35-69	19	**0.0%**	3	**0.0%**	16	**0.0%**
**Age Group**	18-49			557,805	**62.4%**	215,138	**62.5%**	342,667	**62.3%**
	50-64			212,892	**23.8%**	83,658	**24.3%**	129,234	**23.5%**
	65-79			102,517	**11.5%**	38,711	**11.3%**	63,806	**11.6%**
	80+			20,631	**2.3%**	6,448	**1.9%**	14,183	**2.6%**
**Gender**	female			572,502	**64.0%**	200,230	**58.2%**	372,272	**67.7%**
	male			319,698	**35.8%**	143,106	**41.6%**	176,592	**32.1%**
	missing			1,645	**0.2%**	619	**0.2%**	1,026	**0.2%**
**Ethnicity**	African			11,845	**1.3%**	4303	**1.3%**	7,542	**1.4%**
	Another Asian background			10,673	**1.2%**	3,206	**0.9%**	7,467	**1.4%**

	Another Black background	1,540	**0.2%**	615	**0.2%**	925	**0.2%**
	Another ethnic background	6,042	**0.7%**	2138	**0.6%**	3,904	**0.7%**
	Arab	3414	**0.4%**	1198	**0.3%**	2,216	**0.4%**
	Bangladeshi	5,958	**0.7%**	2449	**0.7%**	3,509	**0.6%**
	Caribbean	8,609	**1.0%**	4,009	**1.2%**	4,600	**0.8%**
	Chinese	4,960	**0.6%**	1436	**0.4%**	3,524	**0.6%**
	Indian	30,134	**3.4%**	8,835	**2.6%**	21,299	**3.9%**
	Mixed or multiple ethnic groups	16,517	**1.8%**	6,270	**1.8%**	10,247	**1.9%**
	Pakistani	17,547	**2.0%**	6,524	**1.9%**	11,023	**2.0%**
	Prefer not to say	35,467	**4.0%**	14,110	**4.1%**	21,357	**3.9%**
	White	741,139	**82.9%**	288,862	**84.0%**	452,277	**82.2%**
**NHS Region**	East of England	103,777	**11.6%**	40,107	**11.7%**	63,670	**11.6%**
	London	110,072	**12.3%**	42,238	**12.3%**	67,834	**12.3%**
	Midlands	175,956	**19.7%**	70,125	**20.4%**	105,831	**19.2%**
	North East	138,885	**15.5%**	53,183	**15.5%**	85,702	**15.6%**
	North West	126,282	**14.1%**	47,084	**13.7%**	79,198	**14.4%**
	South East	137,198	**15.3%**	53,217	**15.5%**	83,981	**15.3%**
	South West	101,674		38,001			
	missing	1	**0.0%**	0	**0.0%**	1	**0.0%**
**IMD Quintiles**	1	171,587	**19.2%**	70,469	**20.5%**	101,118	**18.4%**
	2	178,819	**20.0%**	70,693	**20.6%**	108,126	**19.7%**
	3	182,366	**20.4%**	69,992	**20.3%**	112,374	**20.4%**
	4	181,613	**20.3%**	67,913	**19.7%**	113,700	**20.7%**
	5	176,901	**19.8%**	63,932	**18.6%**	112,969	**20.5%**
	Missing	2,559	**0.3%**	956	**0.3%**	1,603	**0.3%**
**Vaccine priority groups**	Heathcare worker	109,776	**12.3%**	20,308	**5.9%**	89,468	**16.3%**
	CEV	81,867	**9.2%**	26,215	**7.6%**	55,652	**10.1%**
	Carehome Resident	2,011	**23.8%**	544	**21.8%**	137,912	**25.1%**
	Immunosuppressed	11,972	**3.2%**	3,931	**1.9%**	8,041	**1.5%**
	At risk	212,862	**0.0%**	74,950	**0.0%**	137,912	**25.1%**
	Tested Positive >90 days previously	28,327	**0.7%**	6,616	**1.9%**	21,711	**3.9%**

* test or onset date from week 37 onwards in those aged 50 years and over with a sample date within 10 days of symptom onset.

Supplementary table 2: Vaccine effectiveness 14 days after booster by interval between second dose and booster, and odds ratio (OR) of testing positive compared to the longest interval between second dose and booster

vaccination status	interval between dose 2 and 3	test negative	test positive	aOR vs 35+ weeks* (95 %CI)	aVE** (95% CI)
unvaccinated	n/a	10322	15481		base
BNT162b2- BNT162b2/ BNT162b2 boost 14+ days ago	25-29 wks	30976	3818	1.54 (1.35-1.76)	93.2 (92.8-93.6)
BNT162b2- BNT162b2/ BNT162b2 boost 14+ days ago	30-34 wks	4052	401	1.32 (1.12-1.56)	94.1 (93.4-94.8)
BNT162b2- BNT162b2/ BNT162b2 boost 14+ days ago ***	35+ wks	3226	272	baseline	95.6 (94.9-96.1)

* model without unvaccinated included ** model with unvaccinated included as baseline *** this group will be those with a short interval between doses 1 and 2
