# Supplementary material for: Effectiveness of COVID-19 booster vaccines against COVID-19-related symptoms, hospitalization and death in England
Source: Nat Med. 2022 Jan 14;28(4):831–7. doi: 10.1038/s41591-022-01699-1 (PMC9018410; doi:10.1038/s41591-022-01699-1)
Supplement: Supplementary file 2 — Reporting Summary [file 41591_2022_1699_MOESM2_ESM.pdf]

nature portfolio

Corresponding author(s): Jamie Lopez Bernal

Last updated by author(s): Jan 11, 2022

Reporting Summary

Nature Portfolio wishes to improve the reproducibility of the work that we publish. This form provides structure for consistency and transparency in reporting. For further information on Nature Portfolio policies, see our Editorial Policies and the Editorial Policy Checklist.

Statistics

For all statistical analyses, confirm that the following items are present in the figure legend, table legend, main text, or Methods section.

n/a	Confirmed	
☐	☒	The exact sample size (*n*) for each experimental group/condition, given as a discrete number and unit of measurement
☒	☐	A statement on whether measurements were taken from distinct samples or whether the same sample was measured repeatedly
☐	☒	The statistical test(s) used AND whether they are one- or two-sided<br>*Only common tests should be described solely by name; describe more complex techniques in the Methods section.*
☐	☒	A description of all covariates tested
☒	☐	A description of any assumptions or corrections, such as tests of normality and adjustment for multiple comparisons
☐	☒	A full description of the statistical parameters including central tendency (e.g. means) or other basic estimates (e.g. regression coefficient) AND variation (e.g. standard deviation) or associated estimates of uncertainty (e.g. confidence intervals)
☐	☒	For null hypothesis testing, the test statistic (e.g. *F*, *t*, *r*) with confidence intervals, effect sizes, degrees of freedom and *P* value noted<br>*Give P values as exact values whenever suitable.*
☒	☐	For Bayesian analysis, information on the choice of priors and Markov chain Monte Carlo settings
☒	☐	For hierarchical and complex designs, identification of the appropriate level for tests and full reporting of outcomes
☒	☐	Estimates of effect sizes (e.g. Cohen's *d*, Pearson's *r*), indicating how they were calculated

Our web collection on statistics for biologists contains articles on many of the points above.

Software and code

Policy information about availability of computer code

Data collection	Custom SQL scripts- Microsoft SQL Server Management Studio 18
Data analysis	Custom SQL and Stata scripts- Microsoft SQL Server Management Studio 18 & Stata 15

For manuscripts utilizing custom algorithms or software that are central to the research but not yet described in published literature, software must be made available to editors and reviewers. We strongly encourage code deposition in a community repository (e.g. GitHub). See the Nature Portfolio guidelines for submitting code & software for further information.

Data

Policy information about availability of data

All manuscripts must include a data availability statement. This statement should provide the following information, where applicable:

- Accession codes, unique identifiers, or web links for publicly available datasets
- A description of any restrictions on data availability
- For clinical datasets or third party data, please ensure that the statement adheres to our policy

This work is carried out under Regulation 3 of The Health Service (Control of Patient Information) (Secretary of State for Health, 2002))(3) using patient identification information without individual patient consent. Data cannot be made publicly available for ethical and legal reasons, i.e. public availability would compromise patient confidentiality as data tables list single counts of individuals rather than aggregated data. Databases used in this study include the National Immunisation Management System (NIMS), Unified Sample Database and the Emergency Care Dataset (ECDS).

Field-specific reporting

Please select the one below that is the best fit for your research. If you are not sure, read the appropriate sections before making your selection.

☐ Life sciences ☒ Behavioural & social sciences ☐ Ecological, evolutionary & environmental sciences

For a reference copy of the document with all sections, see nature.com/documents/nr-reporting-summary-flat.pdf

Behavioural & social sciences study design

All studies must disclose on these points even when the disclosure is negative.

Study description	Test negative case control design that compares the vaccination status in COVID-19 positive and negative cases using quantitative data.
Research sample	English resident population
Sampling strategy	none- whole population of England
Data collection	Routinely collected data: National COVID-19 vaccine register and national COVID-19 testing data. These data are used for clinical management and disease surveillance purposes
Timing	08 December 2020 to 19 November 2021
Data exclusions	Any negative tests taken within 7 days of a previous negative test, or where symptoms were recorded, with symptoms within 10 days of symptoms for a previous negative test were dropped as these likely represent the same episode. Negative tests taken within 21 days before a positive test were also excluded as these are likely to be false negatives. Positive and negative tests within 90 days of a previous positive test were also excluded. Participants contributed a maximum of four randomly chosen negative test results in the follow-up period. Data were restricted to persons who had reported symptoms and gave an onset date. Only persons who had undergone testing within 10 days after symptom onset were included in order to account for reduced sensitivity of PCR testing beyond this period. A small number of positive samples where sequencing was done and they were found not to be the Delta variant were excluded. Finally, only samples taken from 13 September 2021 (week 37, 2021) were retained for analysis.
Non-participation	no participants were involved in the stud
Randomization	No randomization was needed as this is an observational study

Reporting for specific materials, systems and methods

We require information from authors about some types of materials, experimental systems and methods used in many studies. Here, indicate whether each material, system or method listed is relevant to your study. If you are not sure if a list item applies to your research, read the appropriate section before selecting a response.

Materials & experimental systems

n/a	Involved in the study	
☒	☐	Antibodies
☒	☐	Eukaryotic cell lines
☒	☐	Palaeontology and archaeology
☒	☐	Animals and other organisms
☐	☒	Human research participants
☒	☐	Clinical data
☒	☐	Dual use research of concern

Methods

n/a	Involved in the study	
☒	☐	ChIP-seq
☒	☐	Flow cytometry
☒	☐	MRI-based neuroimaging

Human research participants

Policy information about studies involving human research participants

Population characteristics	age (5 year bands), sex, index of multiple deprivation (quintile), ethnic group, care home residence status, geographic region (nhs region), period (calendar week of onset), health and social care worker status, clinical risk group status, clinically extremely vulnerable, severely immunosuppressed, and previously testing positive
Recruitment	Observation study using routinely collected data as part of the UK COVID-19 testing and vaccination programme
Ethics oversight	Surveillance of covid-19 testing and vaccination is undertaken under Regulation 3 of The Health Service (Control of Patient

Ethics oversight	Information) Regulations 2002 to collect confidential patient information (www.legislation.gov.uk/uksi/2002/1438/regulation/3/ made) under Sections 3(i) (a) to (c), 3(i)(d) (i) and (ii) and 3(3). The study protocol was subject to an internal review by the Public Health England Research Ethics and Governance Group and was found to be fully compliant with all regulatory requirements. As no regulatory issues were identified, and ethical review is not a requirement for this type of work, it was decided that a full ethical review would not be necessary.

Note that full information on the approval of the study protocol must also be provided in the manuscript.
