# Supplementary material for: Effectiveness of COVID-19 booster vaccines against COVID-19-related symptoms, hospitalization and death in England
Source: Nat Med. 2022 Jan 14;28(4):831–7. doi: 10.1038/s41591-022-01699-1 (PMC9018410; doi:10.1038/s41591-022-01699-1)
Supplement: Supplementary file 3 — Study protocol. [file 41591_2022_1699_MOESM3_ESM.pdf]

Protocol: Effectiveness of COVID-19 booster vaccines against covid-19 related symptoms, hospitalisation, and death using Pillar 2 community testing data and test negative case-control analysis.

Version 2.2: UK Health Security Agency

1. Background

This is the protocol for the assessing the effectiveness of COVID-19 booster doses in England using community testing through Pillar 2 and the test negative case control design. Many of the methods follow those used recently to assess waning of effectiveness for ChAdOx1-S (Vaxzevria, AstraZeneca) and Pfizer-BioNTech (BNT162b2/ Comirnaty®) vaccines (1).

In the UK, COVID-19 booster vaccines were introduced on 14 September 2021. The UK Joint Committee on Vaccination and Immunisation (JCVI) recommended either a BNT162b2 or a half dose (50µg) of mRNA-1273 (Spikevax, Moderna) vaccine to be given as a booster dose no earlier than 6 months after completion of the primary vaccine course (2).

The initial booster programme is currently for all adults over 50 and those 16-49 years with underlying health conditions that put them at higher risk of severe COVID-19, adult carers and adult household contacts (aged 16 or over) of immunosuppressed individuals, and healthcare workers. On the 30 November 2021 the booster vaccine programme was rolled out to all individual's aged 18 years and over.

2. Objectives

2.1. Aim

To estimate the effectiveness of COVID-19 booster vaccines against covid-19 related symptoms, hospitalisation and death in adults aged 18-49 years and 50 years and older

2.2. Primary objectives

I. Relative VE of each booster vaccine by primary course vaccine type against symptomatic disease compared to 2 doses
II. Relative VE of each booster vaccine by primary course vaccine type against hospitalisation following symptomatic disease compared to 2 doses
III. Relative VE of each booster vaccine by primary course vaccine type against death following symptomatic disease compared to 2 doses

2.3 Secondary objectives

IV. Relative VE compared to the first few days after a booster against symptomatic disease, hospitalisation and death
V. Absolute VE of each booster vaccine by primary course vaccine type against symptomatic disease, hospitalisation and death compared to 0 doses

3. Methods

3.1. Study design

Test-negative case-control design

3.2. Data sources

COVID-19 testing data.

- SARS-CoV-2 Testing Polymerase-chain-reaction (PCR) positive and negative tests from the 08 December 2020 to date of extraction for individuals aged ≥ 18 years from the UKHSA Unified Sample Database.
- Exclude any negative tests taken within 7 days of a previous negative test, or where symptoms were recorded, with symptoms within 10 days of symptoms for a previous negative test (likely represent the same episode).
- Exclude any negative tests within 21 days before a positive test (false negatives)
- Exclude any positive and negative tests within 90 days of a previous positive test
- Participants contribute a maximum of one randomly chosen negative test result in the booster follow-up period.
- Data are restricted to persons who had reported symptoms and gave an onset date.
- Samples must be within 10 days after symptom onset (account for reduced sensitivity of PCR testing beyond this period).
- Exclude any positive samples sequenced and found not to be the Delta variant

After these criteria, as applied samples taken from 13 September 2021 (week 37, 2021) will be retained for analysis.

The National Immunisation Management System (NIMS) demographics

Testing data will be linked to NIMS demographic file to augment the National Health Service number in the testing data using combinations date of birth, surname, first name, and postcode using deterministic linkage with >95.5% uniqueness.

Following this the testing data will be linked using NHS number to the NIMS demographic file to include information on potential confounding variables related to targeted populations: Gender, Ethnicity, NHS region, IMD Quintiles, and vaccine delivery priority groups: healthcare worker, CEV, care home resident, immunosuppression and “at risk” status.

Vaccination data- The National Immunisation Management System (NIMS)

Booster doses will be identified as being a third dose 175 days or more after a second dose and given after 13th September 2021.
Only individuals who are unvaccinated at the time of onset or who have had two or three doses of vaccine at the time of onset will be included. In addition, there must be an interval of at least 175 days from dose2 to dose3 or from dose 2 to onset. Individuals with a mix of vaccines in their primary schedule or less than 19 days between their first and second dose will be excluded. Therefore, a booster is recognised as a booster if given at least 175 days after a second dose.

Emergency Care Data Set (ECDS)

ECDS data include hospital admissions through NHS emergency departments in England but not elective admissions

- Testing data will be linked on NHS number and date of birth to an Emergency Care attendances that have resulted in an inpatient admission.
- Attendances within 14 days of the positive test
- Exclude injury related attendances

Deaths

- Deaths within 28 days of a positive test as recorded in the NIMS data (based on the NHS Patient Demographics Service)

3.3. Study population

All adults resident in England aged 50 years and older reporting symptoms and tested during the study period

3.4. Study period

13 September 2021 (week 37, 2021- vaccine booster roll-out started) to extract date. For hospitalisation and deaths an earlier date for samples included will be used to allow sufficient time for capture of these events.

3.5. Exposure

Vaccination status will be subdivided as follows.

I. Unvaccinated: no vaccine received

II. Primary BNT162b2 course followed by a BNT162b2 booster:
   a. 0-1 days since booster (PF/MD booster combined)
   b. 2-6 since booster (PF/MD booster combined
   c. 7-13 post booster
   d. 14-34 days post booster
   e. 35-69 post booster
   f. 70+days post booster

III. Primary ChAdOx1-S course followed by a BNT162b2 booster:
   a. 0-1 days since booster (PF/MD booster combined)
   b. 2-6 since booster (PF/MD booster combined
   c. 7-13 post booster
   d. 14-34 days post booster
   e. 35-69 post booster

IV. Primary BNT162b2 course followed by a half dose Moderna booster:
   a. 7-13 days since booster

b. 14 days + since booster

V. Primary ChAdOx1-S course followed by a half dose Moderna booster:
  a. 7-13 days since booster
  b. 14 days + since booster

VI. Primary BNT162b2 course given at least 175 days before AND not followed by a booster
VII. Primary ChAdOx1-S course given at least 175 days before AND not followed by a booster

The first few days following the booster could serve as a useful comparator period to estimate the relative VE of the booster to 2 doses as these may reflect a period before the booster has any effectiveness. This may help with possible differences between populations who receive booster doses compared to those who don't. To avoid possible bias due to testing due to any reactogenicity the first 24 hours following the booster may need to be excluded as has been seen with previous doses. The exact days to be used will be assessed visually be plotting the distribution of cases and controls by days since booster. Comparison will only be made within those receiving the same booster.

3.6. Outcomes

- Individuals testing positive for SARS-CoV-2 on PCR who reported symptoms within the preceding 10 days
- Individuals testing positive for SARS-CoV-2 on PCR who report symptoms and have a hospital admission from ECDS within 14 days of testing.
- Individuals testing positive for SARS-CoV-2 on PCR who report symptoms and die within 28 days of testing.

3.7. Data management

Data will be managed using SQL management studio and analysed using Stata. Surveillance of covid-19 testing and vaccination is undertaken under Regulation 3 of The Health Service (Control of Patient Information) Regulations 2002 to collect confidential patient information (www.legislation.gov.uk/uksi/2002/1438/regulation/3/ made) under Sections 3(i) (a) to (c), 3(i)(d) (i) and (ii) and 3(3). The study protocol was subject to an internal review by the Public Health England Research Ethics and Governance Group and was found to be fully compliant with all regulatory requirements. As no regulatory issues were identified, and ethical review is not a requirement for this type of work, it was decided that a full ethical review would not be necessary.

3.8. Sample size and analysis frequency

Analysis will be dose approximately 5 weeks after the booster program has been introduced to allow time for data to accrue to allow good precision of VE estimates. Analysis will be repeated at approximately 3 week intervals and later analyses will allow inclusion of hospitalisations and then deaths. Initial analyses will not include these end points.

3.9. Statistical analysis

We will use a test-negative case-control design to estimate vaccine effectiveness of a booster dose of BNT162b2 vaccine against PCR-confirmed symptomatic disease, hospitalisation and death. Analysis will be by logistic regression with the PCR test result as the dependent variable where those

testing positive are cases and those testing negative controls. Vaccination status will include as an independent variable and effectiveness defined as 1- odds of vaccination in cases/odds of vaccination in controls. Estimates will be reported with 95% confidence intervals. Analyses will stratified by which primary doses had been received.

Vaccine effectiveness will be adjusted in logistic regression models for:

- age (5 year bands)
- sex
- index of multiple deprivation (quintile)
- ethnic group
- care home residence status
- geographic region (nhs region)
- period (calendar week of onset)
- health and social care worker status
- clinical risk group status
- clinically extremely vulnerable
- severely immunosuppressed
- previously testing positive

These factors will all be considered potential confounders so will be included in all models.

Primary analysis

Boosters will be compared to two primary doses with at least 175 days prior to the onset but with no booster dose recorded. The comparison will be within those with the same priming vaccine.

Secondary analysis

- Boosters compared to unvaccinated individuals (this will also allow assessment of residual effectiveness of 2 doses).
- Boosters compared to a period shortly after a booster dose was received-The period will be selected after plotting the data on case and control numbers after the booster dose and to avoid vaccine reactogenicity which may affect the case-control ratio

4. Limitations

- Imperfect sensitivity PCR testing could cause misclassification of both cases and controls
- Many individuals will also have been previously infected so the VE measured is in the context of a population where many have already had natural exposure
- Unaccounted residual confounding
- The unvaccinated as a comparator is most susceptible to residual confounding as the totally unvaccinated population may differ in many ways to those who have had vaccine doses, many of which may lead to underestimation of VE. If a period shortly after dose 3 can be identified this should have the least issues with confounding.

5. Reporting

Reports will be produced for JCVI and made available on MEDRXIV before preparation for publication in a peer reviewed journal.

All reports will also be published here: https://www.gov.uk/guidance/monitoring-reports-of-the-effectiveness-of-covid-19-vaccination

6. Additional exploratory analyses

15/11/2021: Additional analysis planned to assess VE according to the interval between the first and second dose. Intervals to be determined after examination of the distribution of intervals by age and vaccine. Comparison will be of absolute VE against unvaccinated by interval and of odds of testing positive 14 or more days after a booster in those with shorter intervals compared to longer intervals.

7. Changes to the analysis plan between version 2.1 (the final version before the first booster analysis) and 2.2 (current version)

Change	date	Reason
Addition of 18-49 analysis	06/12/2021	This age group was added to general recommendation for boosting
Maximum of 1 test negative in the follow-up period	06/12/2021	As the booster period is quite short using up to 4 random negatives is not necessary and using 1 helps ensure independence.
Addition of post booster intervals of 14-34,35-69 and 70+ days post vaccination	06/12/2021	More time has now passed to allow potential waning to be assessed.
Change from 140+ days to 175+ days as the comparator follow-up period post dose 2	06/12/2021	The vast majority of boosters given at the time of the study were from 175+ days so this represents a better estimate of the waned post dose 2 level of protection.

References

1. Andrews N, Tessier E, Stowe J, Gower C, Kirsebom F, Simmons R, et al. Vaccine effectiveness and duration of protection of Comirnaty, Vaxzevria and Spikevax against mild and severe COVID-19 in the UK. MedRxiv. 2021:2021.09.15.21263583.

2. Department of Health & Social Care. JCVI statement regarding a COVID-19 booster vaccine programme for winter 2021 to 2022 [Available from: https://www.gov.uk/government/publications/jcvi-statement-september-2021-covid-19-booster-vaccine-programme-for-winter-2021-to-2022/jcvi-statement-regarding-a-covid-19-booster-vaccine-programme-for-winter-2021-to-2022.
